# Alveolar Macrophage Innate Response to *Mycobacterium immunogenum*, the Etiological Agent of Hypersensitivity Pneumonitis: Role of JNK and p38 MAPK Pathways

**DOI:** 10.1371/journal.pone.0083172

**Published:** 2013-12-11

**Authors:** Harish Chandra, Ekta Yadav, Jagjit S. Yadav

**Affiliations:** Microbial Pathogenesis Laboratory, Department of Environmental Health, University of Cincinnati College of Medicine, Cincinnati, Ohio, The United States of America; French National Centre for Scientific Research, France

## Abstract

*Mycobacterium immunogenum* is an emerging pathogen of the immune-mediated lung disease hypersensitivity pneumonitis (HP) reported in machinists occupationally exposed to contaminated metal working fluid (MWF). However, the mechanism of its interaction with the host lung is unclear. Considering that alveolar macrophages play a central role in host defense in the exposed lung, understanding their interaction with the pathogen could provide initial insights into the underlying immunopathogenesis events and mechanisms. In the current study, *M. immunogenum* 700506, a predominant genotype isolated from HP-linked fluids, was shown to multiply intracellularly, induce proinflammatory mediators (TNF-α, IL-1α, IL-1β, IL-6, GM-CSF, NO) and cause cytotoxicity/cell death in the cultured murine alveolar macrophage cell line MH-S in a dose- and time-dependent manner. The responses were detected as early as 3h post-infection. Comparison of this and four additional genotypes of *M. immunogenum* (MJY-3, MJY-4, MJY-12, MJY-14) using an effective dose-time combination (100 MOI for 24h) showed these macrophage responses in the following order (albeit with some variations for individual response indicators). Inflammatory: MJY-3 ≥ 700506 > MJY-4 ≥ MJY-14 ≥ MJY-12; Cytotoxic: 700506 ≥ MJY-3 > MJY-4 ≥ MJY-12 ≥ MJY-14. In general, 700506 and MJY-3 showed a more aggressive response than other genotypes. Chemical blocking of either p38 or JNK inhibited the induction of proinflammatory mediators (cytokines, NO) by 700506. However, the cellular responses showed a somewhat opposite effect. This is the first report on *M*. *immunogenum* interactions with alveolar macrophages and on the identification of JNK- and p38- mediated signaling and its role in mediating the proinflammatory responses during these interactions.

## Introduction

Hypersensitivity pneumonitis (HP), an immunologically mediated alveolar and interstitial lung pathology, is an occupational disease that has been reported among machinists for more than a decade [1]. HP-associated metal working fluids (MWF) used in automotive plants and other machining operations have revealed predominant growth of non-tuberculous mycobacteria (NTM) of the *M. chelonae-M. abscessus* complex. These mycobacterial species have been implicated in HP [2], [3], among other respiratory symptoms in the exposed machinists. Particularly, *Mycobacterium immunogenum* (MI), a recently discovered member of this species complex, has been linked with occupational HP in workers exposed to contaminated MWF (from which this species has frequently been cultured [4], [5]). Subsequently, multiple genotypes of *M. immunogenum* have been isolated from diverse MWF operations in our recent efforts [6], [7]. While *M. immunogenum* is considered as the etiological agent of the MWF-associated HP based on human epidemiological [8], [9], [10] and animal exposure studies [11], [12], the exact mode of interaction of this pathogen with the exposed lung remains unclear. Also, the relative pathogenesis potential of individual genotypes of this pathogen prevalent in the occupational settings is not known.

In general, the outcome of lung exposure to respiratory bacterial pathogens is believed to be the net consequence of the innate and adaptive immune defenses of the host and a pathogen’s capacity to subvert them. It is well known that alveolar macrophages play a central role in regulating the innate and acquired immune responses against pathogens. Moreover, alveolar macrophages are largely considered to be the preferential site for bacterial killing or proliferation thereby generating the antigen load of the pathogen in human lung tissue [13]. Mycobacteria, in general, activate both humoral and cell-mediated immune responses in other infections [14], [15]. However, the mechanism(s) by which *M. immunogenum* interaction occurs in the lung destined for HP development is not yet clear. Given that HP is a cell-mediated immune disorder, it may be assumed that innate activation of macrophages and development of cell-mediated immunity is critical in this disease process. The regulation of key cytokines by alveolar macrophages is considered one of the important immune regulatory functions during development of T-helper cell phenotypes. However, the pattern of expression of these mediators in *M. immunogenum* interactions with alveolar macrophages has not yet been reported. Hence, understanding the alveolar macrophage response to *M. immunogenum* infection will pave the way for understanding the pathogenesis mechanisms of mycobacterial HP.

In host-pathogen interaction, different strains or variants (genotypes or morphotypes) of a pathogen might show differential pathogenesis by responding differently in terms of intracellular survival/growth and induction of host response. Understanding these differences may allow understanding of the basis of virulence potential of individual strains and the responsible virulence factors. Since practically nothing is known about the relative virulence/immunogenic potential of *M. immunogenum* strains/variants, we compared five *M. immunogenum* genotypes, originally isolated in our previous efforts, for their interaction with alveolar macrophages.

Considering that no information is available on the signaling mechanisms underlying the lung inflammatory response in HP, it is significant that this study demonstrates contribution of MAP kinase-mediated signaling in alveolar macrophage activation and response caused by *M. immunogenum*. To our knowledge, this marks the beginning of understanding of the signaling mechanisms of host-pathogen interaction in this emerging pathogen.

## Materials and Methods

### Strains and culture conditions

*Mycobacterium immunogenum* genotypes 700506, MJY-3 MJY-4, MJY-12 and MJY-14, originally isolated from diverse contaminated metal working fluids [7], [5] were maintained by sub culturing on Middlebrook 7H10 agar or in Middlebrook 7H9 broth (Difco Laboratories, Sparks, MD, USA) and storing as frozen glycerol stocks in the same medium. Each genotype was grown to mid-log phase in Middlebrook 7H9 broth (Difco Laboratories, Sparks, MD, USA) supplemented with 10% Oleic acid-Albumin-Dextrose-Catalase (OADC) enrichment medium (BD Biosciences, Sparks, MD, USA) and 0.5% glycerol with continuous shaking (150 rpm) at 37°C to a 150 Klett reading (equivalent to approximately 10^9 ^cfu/ml) measured by using a Klett photoelectric colorimeter (Klett, New York, NY, USA). Shaking reduced the cell clumping thereby facilitating the subsequent procedure for generation of a monodispersed cell suspension (see details below).

### Alveolar macrophages

Murine alveolar macrophage cell line MH-S (CRL-2019), purchased from the American Type Culture Collection (ATCC), Manassas, VA, USA, and was used. The MH-S cells were maintained in RPMI 1640 medium (ATCC, Manassas, VA, USA) supplemented with 10% fetal bovine serum and 1% streptomycin-penicillin-glutamate solution (Invitrogen, Carlsbad, CA, USA). Cells were grown for 48h at 37°C in a humidified 5% CO_2_ incubator, and were collected, counted, and adjusted to a concentration of 1×10^6^ cells/ml for further use. The alveolar macrophage cells were seeded in 12-well culture clusters at a density of 1×10^6^ cells/well 12 hours prior to the bacterial challenge.

**Preparation of single-cell suspensions of** ***M. immunogenum*** **genotypes for infection** For all macrophage infection experiments with *M. immunogenum* 700506, single cell suspension for use as inoculum was prepared as follows. A liquid culture (50 ml) grown to mid-log phase (150 Klett reading) was centrifuged at 3000 x g for 15 min and the cell pellet resuspended in 10 ml of complete RPMI medium (without antibiotics). The suspension was passed serially (ten times) through a 20 gauze syringe needle using a glass syringe and then through a 25 gauze syringe needle. Remaining small clumps were removed with additional centrifugation at 350 × g for 5 min. The resulting single cell suspension, confirmed based on microscopy, was quantified (colony forming units/ml) by spread plate method using Middlebrook 7H10 agar (supplemented with OADC). A freshly prepared inoculum prepared using this optimized protocol was used for macrophage infections. For genotype comparison experiments, individual genotypes were first grown on 7H10 agar-OADC plates and 10 loopfuls of bacterial cells from each plate were separately resuspended in 10 ml of complete RPMI medium and single cell suspensions were prepared by serial passaging through syringe needles as described above. All inocula were normalized to 10^8^ cfu/ml before use in infection experiments.

### Experimental design

*M. immunogenum* genotype 700506 was used as a reference strain to investigate the effective dose- and time- of exposure. The selected dose-time combination was then used for comparing the different genotypes. The freshly propagated MH-S cells, first allowed to adhere as monolayers in 12-well culture clusters (1×10^6^ cells/well), were infected with the 700506 inoculum at varying multiplicity of infection (MOI), ranging from .001 (10^3^ cfu/10^6^ MH-S cells) to 1000 (10^9^ cfu/10^6^ MH-S cells) MOI. MH-S cells stimulated with lipopolysaccharide (LPS, Sigma Aldrich, St. Louis, MO) @ 500 ng/ml served as positive control for assay validations. The MH-S cells treated with an equal volume of the vehicle (antibiotic-free RPMI medium) were used as negative control. Each treatment was carried out in triplicate. Subsequently, the experimental 12-well cultures challenged with the selected effective infection dose were incubated at 37°C in a humidified 5% CO_2_ incubator for varying periods of time (3h, 6h, 12h and 24h). At each time point, the treated cells were harvested and subjected to further analyses. The selected effective pathogen dose and time combination was then used to compare the other four genotypes (MJY-3, MJY-4, MJY-12 and MJY-14).

In an independent experiment, the MH-S cells were treated with MAP kinase inhibitors, using two different doses (20 µM and 40 µM) of the p38 inhibitor SB202190 (EMD Biosciences, San Diego, CA), or the JNK inhibitor SP600125 (EMD Biosciences, San Diego, CA), 1h prior to the challenge with *M. immunogenum* 700506. Positive (no-inhibitor) and negative (vehicle) controls were run in parallel for comparison. Macrophage cells were harvested at 24h post-exposure and subjected to further analysis, as described below.

### Cytotoxicity

Cytotoxicity in the pathogen-challenged MH-S cells was measured in terms of release of lactate dehydrogenase (LDH) using Cyto Tox96 non-radioactive cytotoxicity assay kit (Promega, Madison, WI, USA) per manufacturer’s instructions.

### Cell viability

Macrophage cell viability changes were estimated using trypan blue staining (Invitrogen, Carlsbad, CA, USA) for microscopic counting of the live and dead cells using hemocytometer (Bausch & Lomb Rochester, NY USA).

### Nitric oxide (NO)

The levels of NO in the culture supernatants were estimated as nitrite using the Griess reagent system per manufacturers’ instructions (Promega, Madison, WI, USA).

### Measurement of cytokines by ELISA

Levels of different cytokines (TNF-α, IL-6, 1L-1α, IL-1β, and IL-10 and GM-CSF) were measured in the culture supernatants or cell lysates from macrophage monolayers infected with *M. immunogenum* genotypes. Sandwich ELISA kits were used to detect the cytokine levels, following the manufacturer’s protocol and instructions (eBioscience, San Diego, CA, USA).

### Determination of intracellular pathogen replication

MH-S cells (1×10^6^ cells/well) were adhered for 4 hours followed by washing (three times) with RPMI medium-without-antibiotics (RPMI-A). The cells were infected with MI 700506 at 10 multiplicity of infection (MOI) for 1hour, followed by washing (3x) with RPMI-A medium and incubation in presence of gentamycin (10 µg/ml) for 30 min. to kill extracellular bacteria in the medium. The infected cells were again washed (3x) followed by incubation in RPMI-A medium. Efficacy of the gentamycin treatment in terms of complete inactivation of the extracellular bacteria was confirmed by plating the culture supernatant on Sauton’s agar. For determination of intracellular pathogen load, the cells were lysed at different time points of incubation, using SDS (0.25%) and neutralized using bovine albumin (0.1%). Serial dilutions of the lysate were spread plated on Sauton’s agar for determination of the intracellular pathogen load in terms of colony forming units (CFUs).

### Transmission electron microscopy

MI 700506-infected (24 hours) and uninfected (control) MH-S cells were washed twice in 0.175 M sodium cacodylate buffer (pH 7.4) and fixed using 3% gluteraldehyde. The fixed cells were processed for transmission electron microscopy at the Cincinnati Children’s Hospital Medical Center Pathology Research Core facility, using their standard procedures. Images were taken at a magnification of 30,000 X using AMT digital camera system (Advanced Microscopy Techniques, Corp., Woburn, MA, USA).

### Western blot analysis

MH-S cells (1×10^6^/well) adhered for 4 hours in a 12-well plate were infected with MI 700506 for 60 min. using various MOIs, ranging from 0.001 to 1000. The infected cells were washed (3x) with RPMI-A and lysed with RIPA buffer containing a protease and phosphatase inhibitors cocktail. Western blot analysis was performed using the primary antibodies anti-phospho p38, anti-p38, anti-phospho SAPK/JNK, and anti-SAPK/JNK (all from Cell Signaling Technology, Boston, MA, USA), each at a dilution of 1∶1000. Anti-β Actin was used at a dilution of 1∶10,000 (Sigma, USA). HRP-conjugated anti-rabbit and anti-mouse secondary antibodies (Cell Signaling Technology, Boston, MA, USA) were used at 1∶1000 and 1∶2000 dilutions, respectively. The bands were visualized with an ECL kit per manufacturer’s instructions (Pierce Chemical, Rockford, IL, USA) and subjected to densitometric quantification using the NIH Image J software.

### Statistical analysis

The data are presented as means ± standard deviation (SD) obtained from at least three independent experiments, each performed in triplicate. Differences between groups were assessed by the paired two-tailed Student’s t test with level of significance P≤0.05 (n = 9) being accepted as statistically significant.

## Results

### 1. Dose- and time- dependent responses of alveolar macrophages to MI exposure

Alveolar macrophages infected with MI 700506 showed dose- and time-dependent immunological and cellular responses.

**Figure 1:**
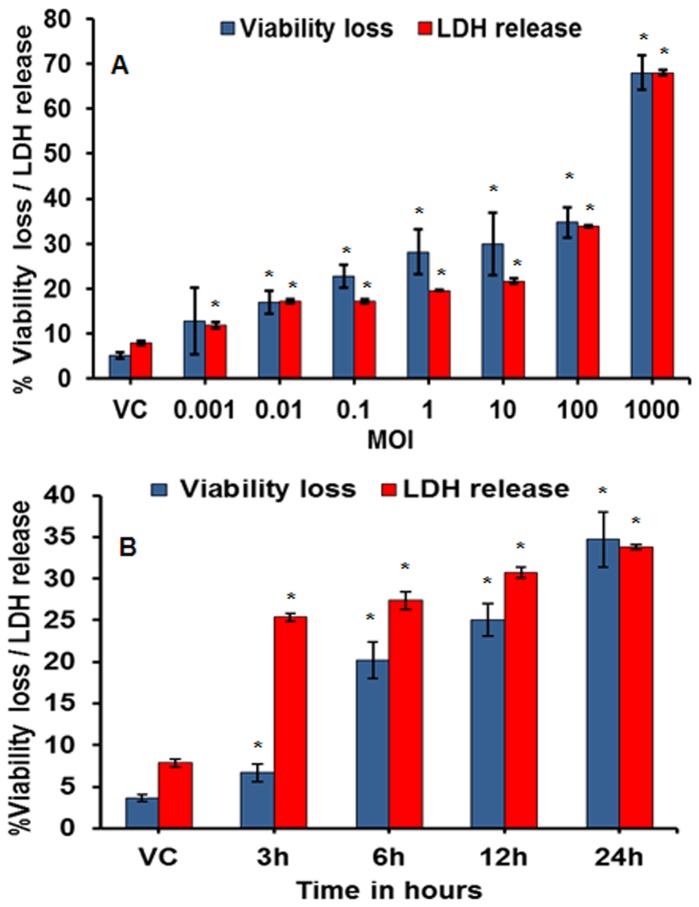
Induction of cellular responses (in terms of LDH release and cell death) in the MH-S cells after 24h exposure to *M. immunogenum* 700506. A. Effect of varying the infection dose. B. Effect of time course after exposure to a selected infection dose (100 MOI). Values presented as means ± standard deviations are from three independent experiments; each experiment was done in triplicate. Asterisk (*) indicates statistically significant (*P*≤0.05) difference as compared to the vehicle control.

**Macrophage cytotoxicity and cell death.** LDH release, an indirect indicator of cytotoxicity, due to the loss of cell membrane integrity or cell lysis, increased in a dose- and time- dependent manner in the MH-S cells infected with MI 700506. The LDH release (Fig 1A) began at 0.001 MOI and continued to show a steady increase with dose through 100 MOI. This was followed by a more dramatic increase in the next higher dose (1000 MOI). The more direct assay for viability loss (Trypan blue exclusion-based assay) yielded a dose-response pattern quite parallel to the one followed by the LDH release (Fig 1A). Time-course exposure (3h to 24h) of the MH-S cells with 100 MOI (a dose that gave a reasonably high measurable response) showed a significant induction of cytotoxicity as early as 3h which continued through 24h post-infection (Fig 1B). In terms of cell viability, the selected dose caused a slight loss at 3h post-infection but a significant loss after 6h of infection, which continued to increase through 24h post-infection (Fig 1B). This showed that onset of LDH increase preceded the onset of viability loss in the infected macrophages.

**Figure 2:**
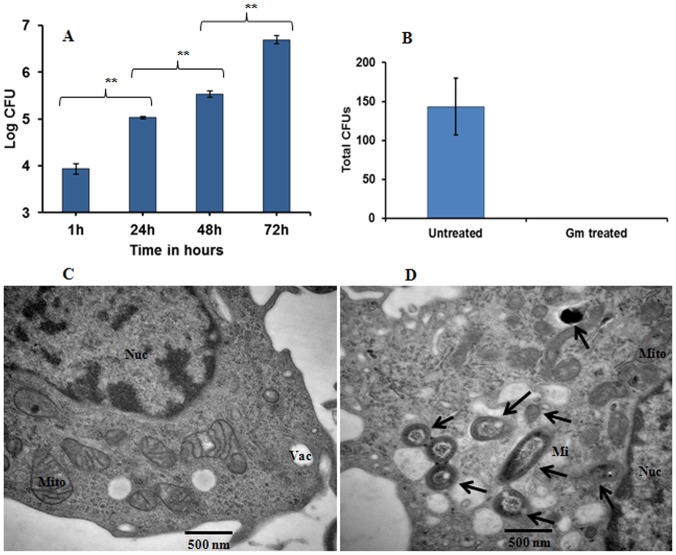
Determination of intracellular replication of *M. immunogenum* 700506 in alveolar macrophages (MH-S cells). **A.** Cells infected with bacteria for 1 hour were made free of extracellular bacteria by repeated (3x) washing followed by gentamycin treatment as described under Materials and Methods section and their intracellular bacterial load was monitored for 72 hours based on CFU analysis. **B.** Inactivation of extracellular bacteria in gentamycin treatment. Log CFU values presented as means ± standard deviations are from three independent experiments; each experiment was done in triplicate. Asterisk (**) indicates statistically significant (*P*≤0.01) difference as compared to the values for other time points. **C& D.** Transmission electron microscopy (TEM) images of uninfected (C) and MI-infected (D) cells at a magnification of 30000X. Black arrows indicate the intracellular *M. immunogenum* cells. Abbreviations: Gm (gentamycin), Mi (*M. immunogenum*), Nuc (nucleus), Vac (vacuole), and Mito (mitochondrion), respectively.

**Intracellular replication of the pathogen**. The fate of *M. immunogenum* inside the macrophages is not known. To examine whether MI survives and replicates inside the alveolar macrophages post-phagocytosis (1 hour), we followed infection dynamics for 72 hours. The test strain showed a steady increase in intracellular multiplication leading to a 2.6 log increase in pathogen load (Fig 2A). The measured bacterial load was a result of the replicating intracellular bacteria, as gentamycin was shown to completely inactivate all extracellular bacteria (Fig 2B). Direct evidence for the intracellular presence of MI was obtained by Transmission electron microscopy on 24h-infected cells wherein MI was seen localized mostly in vacuoles (Fig 2C and D). We also observed indication of the presence of intracellular bacteria in the macrophage cells by acid fast staining method (data not shown).

**Figure 3:**
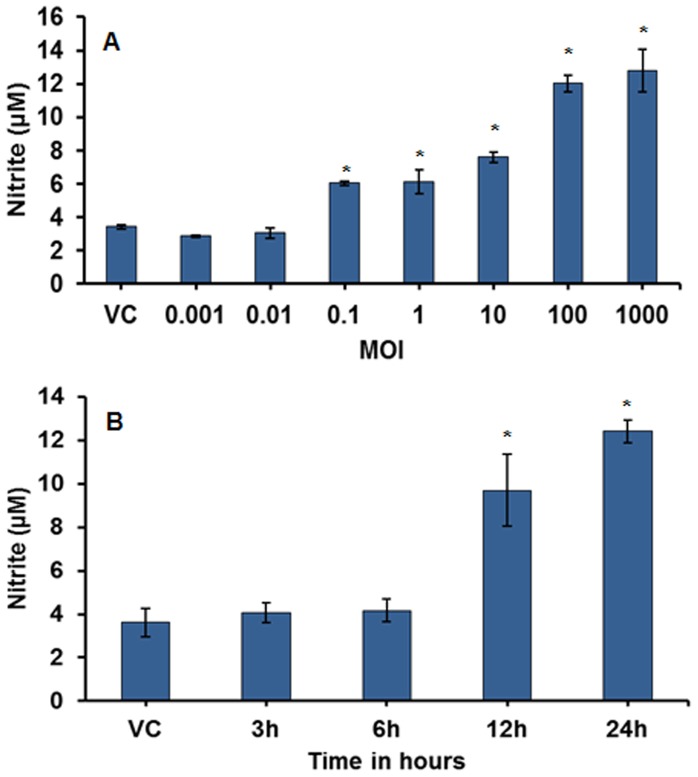
Induction of nitric oxide (NO) production (measured as nitrite) in MH-S cells after 24h exposure to *M. immunogenum* 700506. A. Effect of varying the infection dose. B. Time course of NO production after exposure to a selected dose (100 MOI). Values are presented as means ± standard deviations calculated based on the data obtained from three independent experiments; each experiment was done in triplicate. Asterisk (*) indicates statistically significant (*P*≤0.05) difference as compared to the vehicle control.

**NO Production**. Extracellular NO production increased in a dose- and time- dependent manner compared to the vehicle control (3.42±0.104 µM). In the dose-response experiment (Fig 3A), there was a significant (p≤0.05) increase in NO production at doses 0.1 MOI (6.04±0.105 µM) through 1000 MOI (12.78±1.25 µM). The increase was more pronounced and comparable at the high doses (100 and 1000 MOI). In the time-course analysis (Fig 3B), the 100 MOI dose resulted in a significant increase in NO induction beginning at the 12h time-point and continuing through 24h post-infection. Unlike cytotoxicity/cell vitality loss, no significant induction was observed at 3h and 6h post-infection, as compared to the vehicle control.

**Figure 4:**
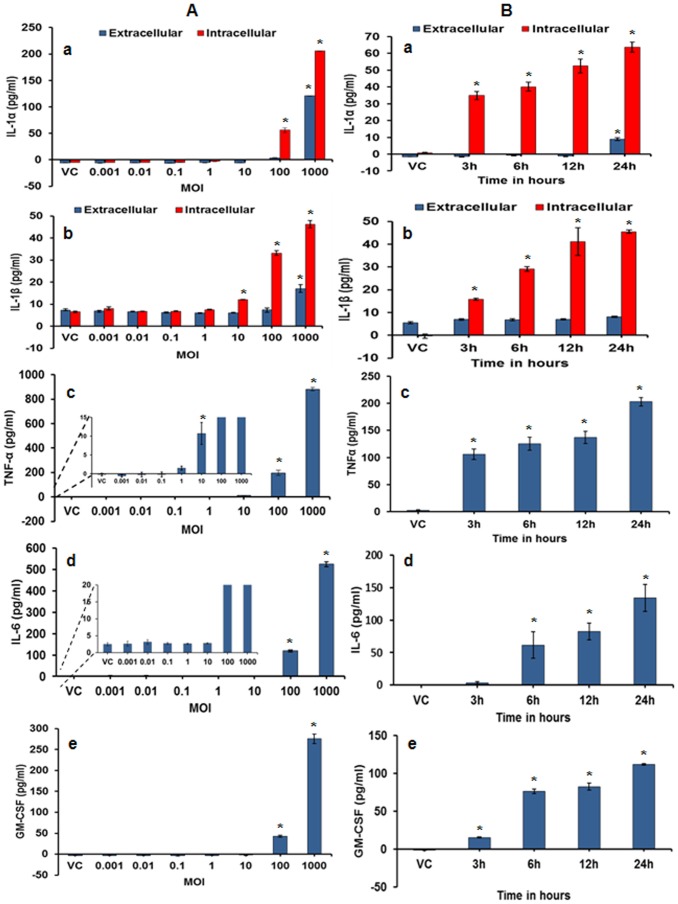
Induction of cytokine response in MH-S cells after 24h post-exposure to *M. immunogenum* 700506. **A.** Effect of varying the infection dose on the expression of different cytokines in MH-S cells at 24h post-infection (**a**) IL-1α (**b**) IL-1β, (**c**) TNF-α, (**d**) IL-6, (**e**) GM-CSF. **B.** Time course of expression of different cytokines in MH-S cells exposed to an infection dose of 100 MOI (**a**) IL-1α (**b**) IL-1β, (**c**) TNF-α, (**d**) IL-6, (**e**) GM-CSF. Values are presented as means ± standard deviations calculated based on the data obtained from three independent experiments; each experiment was done in triplicate. A statistically significant (*P*≤0.05) value as compared to vehicle control is indicated by an asterisk (*).

**Expression of cytokines.** The proinflammatory cytokines TNFα, IL-1β, IL-1α, and IL-6 and GM-CSF were found to be upregulated in a dose- and time- dependent manner whereas the anti-inflammatory cytokine IL-10 was not detected within or outside the cells in the infected macrophage cultures (Fig 4). Similar to the cellular responses, all upregulated proinflammatory cytokines showed an increase in expression with the increase in pathogen dose and the effect was significant at 100 and 1000 MOI (Fig 4A subpanels a through e). In terms of the production kinetics, majority of the inflammatory cytokines were detected as early as 3h post-infection and increased gradually with time reaching a maximum at the highest time point (24h) post-infection (Fig 4B- subpanels a through e). Interestingly, IL-1β and IL-1α were detected primarily in cell lysates, indicating their intracellular localization (Fig 4B- subpanels a and b). Significant induction (p≤0.0012) in IL-1α production was also detected extracellularly but only at 24h post-infection at the highest doses (100 and 1000 MOI).

### 2. Comparison of MI genotypes for macrophage response

The preceding MI 700506 experiments allowed us to select an effective and realistic dose-time combination (100 MOI for 24h) based on induction of cellular and immunological responses in MHS cells. Using this dose (100 MOI) and exposure time (24h) combination, both inflammatory and cellular damage responses were compared for the five different MI genotypes, namely 700506, MJY-3, MJY-4, MYJ-12 and MJY-14.

**Figure 5:**
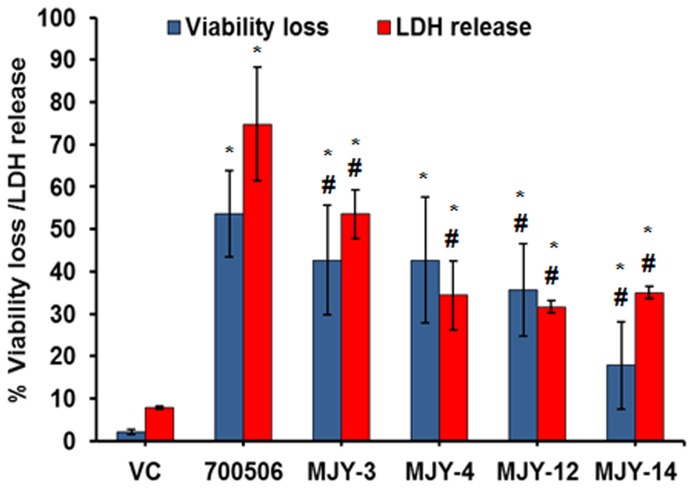
Comparison of *M. immunogenum* genotypes 700506, MJY-3, MJY-4, MJY-12 and MJY-14 for induction of cellular response in terms of LDH release and cell viability loss in MH-S cells. The cells were infected at a 100 MOI dose for 24h. Values are presented as means ± standard deviations calculated based on the data obtained from three independent experiments; each experiment was done in triplicate. Asterisk (*) indicates statistically significant (*P*≤0.05) difference from the Vehicle-treated group and pound sign (#) indicates statistically significant (*P*≤0.05) difference from the 700506-treated group.

Of these, the 700506 genotype gave the highest cellular response. MJY-3 increased LDH release (up to 53.4±5.66%) to significantly (p≤0.0057) higher levels as compared to the other genotypes next only to the 700506 genotype (74.8±13.4%). This cytotoxicity trend paralleled with the observed cell viability loss in the MJY-3 treated group (42.7±12.84 percent) as compared to 700506 (53.63±10.30%) and other genotypes (Fig 5). These results demonstrate that MJY-3 is the most potent among the other four genotypes in terms of inducing a cellular response in alveolar macrophages.

**Figure 6:**
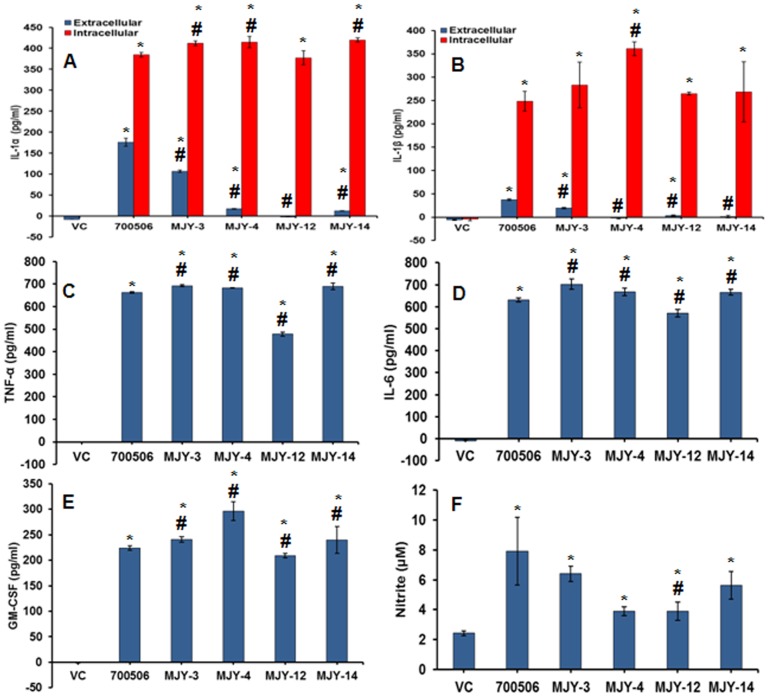
Comparison of *M. immunogenum* genotypes 700506, MJY-3, MJY-4, MJY-12 and MJY-14 for induction of proinflammatory mediators (cytokines/NO) in MH-S cells. The cells were infected at a 100 MOI dose for 24h. A & B. Levels of proinflammatory cytokines IL-1α and IL-1β in both culture supernatant and cell lysate. C, D, & E. Levels of TNF- α, IL-6 and GM-CSF in cell culture supernatant. F. Level of NO measured as nitrite in cell culture supernatant. Asterisk (*) and pound sign (#) indicate statistically significant (*P*≤0.05) difference from the vehicle-treated and 700506-treated groups, respectively. Values are presented as means ± standard deviations calculated based on the data obtained from three independent experiments; each experiment was done in triplicate.

The individual genotypes showed significant but differential response in terms of induction of proinflammatory mediators (cytokines/NO) in the MH-S cells (Fig 6 A-F). In terms of cytokine expression, MJY-3, MJY-4, and MJY-14 showed relatively higher responses than 700506 whereas MJY-12 showed the lowest response. For NO induction, MJY-3 induced the highest amount of NO (6.4±0.5 µM), next only to 700506, as compared to the other genotypes (MJY-14, MJY-4, and MJY-12). Collectively, the results imply that MJY-3 and 700506 are the most potent genotypes for inducing inflammatory and cellular damage responses in alveolar macrophages.

### 3. Role of MAPK signaling in macrophage response to *M. immunogenum*

**Figure 7:**
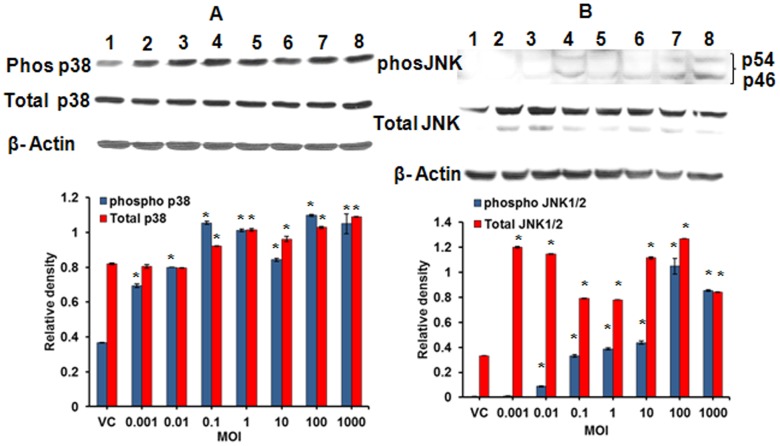
Activation of MAPKs in alveolar macrophages on infection with *M. immunogenum* 700506. **A.** p38 immunoblot. **B.** SAPK/JNK immunoblot. Activation was assessed in terms of upregulation of total MAPK expression levels and increase in phosphorylated MAPK. Densitometric analysis of the Western blots was done using the NIH’s Image J software. **Lanes 1-8** represent vehicle control (VC), 0.001, 0.01, 0.1, 1, 10, 100 and 1000 MOI, respectively. Details on the antibodies for total- and phospho- MAPKs and β-actin are described under the Materials and Methods section. Values are presented as means ± standard deviations calculated based on the data obtained from three independent experiments; each experiment was done in triplicate. Asterisk (*) indicates statistically significant (*P*≤0.05) difference as compared to the vehicle control.

Considering the highest potential of MI 700506 for induction of cellular and immunological responses in MH-S cells, we chose this genotype to examine the role of MAPK pathway signaling in cytotoxicity/cell death and inflammatory response (cytokine/NO expression). Infection of MH-S cells with increasing pathogen dose (0.001 through 1000 MOI) for 1 hour upregulated the total p38 and JNK levels and efficiently phosphoactivated JNK and p38 in a dose-dependent manner, as evident from the immunoblots obtained using total- and phospho- p38 (Thr180/Tyr182) and total- and phospho-JNK( Thr183/Tyr185) specific antibodies (Fig 7A and B). Unlike JNK expression levels which were upregulated significantly at all infection doses, p38 levels required a minimum infection dose of 0.1 MOI for its upregulation. The dose-response for JNK upregulation however showed a non-linear trend unlike p38 which showed an almost linear dose-dependent trend (Fig 7A). The phosphoactivation did not follow the same trend as the upregulation (Fig 7B). For instance, p38 was phosphoactivated at all doses whereas JNK was activated beginning 0.01 MOI. A noticeable difference in p38 and JNK was the differential effect of the highest infection dose (1000 MOI); while p38 showed almost similar effect of 100 and 1000 MOI, JNK showed a decrease in levels and activation at the highest dose (1000 MOI).

**Figure 8:**
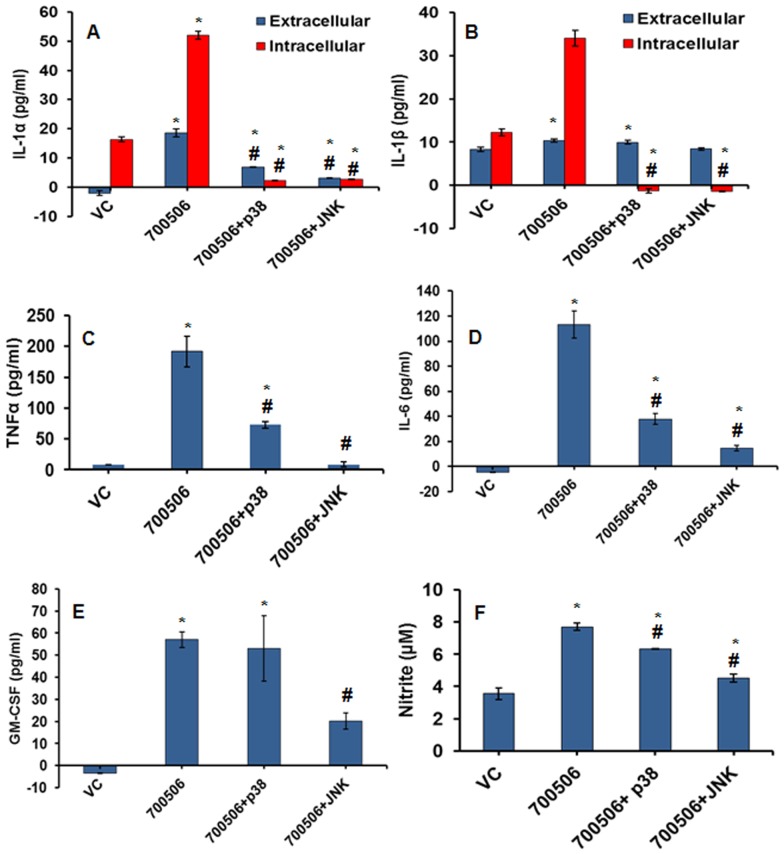
Effect of p38 inhibitor (SB202190) and JNK inhibitor (SP600125) on expression of proinflammatory mediators (cytokines/NO) in MH-S cells infected with *M. immunogenum* 700506. A & B. Levels of proinflammatory cytokines IL-1α and IL-1β in cell culture supernatant and lysate. C, D, & E. Levels of TNF-α, IL-6, and GM-CSF in cell culture supernatant. F. Levels of NO production (measured as nitrite). Values are presented as means ± standard deviations calculated based on the data obtained from three independent experiments; each experiment was done in triplicate. Asterisk (*) and pound sign (#) indicate statistically significant (P≤0.05) difference from the vehicle control and 700506 (no inhibitor) control, respectively.

**Figure 9:**
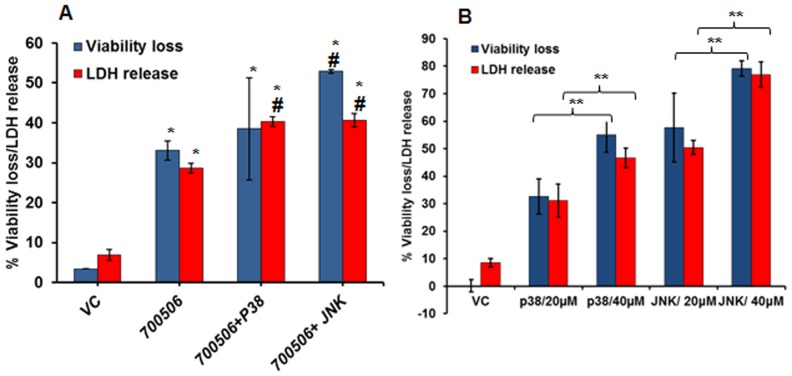
Effect of MAPK inhibitors on cellular responses (in terms of Cell viability and LDH release) in MH-S cells infected with *M. immunogenum* 700506. A. Demonstration of the effect of the p38 inhibitor (SB202190) and JNK inhibitor (SP600125), each at 20 µM concentrations, in comparison with the no-inhibitor control (positive control) and vehicle control (negative control). Asterisk (*) and pound sign (#) indicate statistically significant (P≤0.05) difference from the vehicle control and 700506 (no inhibitor) control, respectively. B. Dose-dependent effect of p38 inhibitor (SB202190) and JNK inhibitor (SP600125) on the cellular responses using 20 µM and 40 µM concentrations. Asterisks (**) indicate statistically significant (P≤0.001) difference between different concentrations of inhibitor. Values are presented as means ± standard deviations calculated based on the data obtained from three independent experiments; each experiment was done in triplicate.

Alternately, the MH-S cells were pretreated with p38 and JNK inhibitors to understand the role of MAPKs based on inhibition. Interestingly, use of a p38 inhibitor (SB202190) or a JNK inhibitor (SP600125) 1h prior to challenging with 700506 led up to > 80% (p≤0.0051) decrease in the tested cytokines including IL-1α, IL-1β, TNF-α, IL-6, and GM- CSF (Fig 8A to E) as well as a significant inhibition of NO induction (Fig 8F). On the other hand, the two inhibitors showed a somewhat opposite effect on cellular damage response (Fig 9A); the effect was further confirmed using increasing doses of the inhibitors (Fig 9B). Taken together, the MAPK inhibition (p38 and JNK) abrogated in part the proinflammatory response suggesting the role of MAPK pathway in HP immunopathogenesis.

## Discussion

HP, also known as an extrinsic allergic alveolitis, is primarily an immune-mediated disorder which is characterized by inflammation changes in the lung. Alveolar macrophages (AMs) are the first line of defense against any invading bacterial pathogens in the exposed lung and are known to mediate both innate and adaptive immune responses. AMs offer a hostile environment to some pathogens and a conducive environment for survival/multiplication to others, the latter including the species of pathogenic mycobacteria such as *M. tuberculosis*. However, the fate of *M. immunogenum* inside the macrophages *per se* is not yet known. The current study being the first attempt to understand the survival and/or multiplication of this relatively new mycobacterial pathogen is therefore significant. Based on multiple lines of evidence (intracellular CFU changes, electron microscopy), our data show that MI is indeed capable of intracellular survival and multiplication within alveolar macrophages and shows significant rate of buildup of pathogen load (2.6-log in 72 hours). Alveolar macrophages were therefore used as a surrogate to study host lung-pathogen interactions of *M*. *immunogenum* in this study. As the study involved the use of a range of variables and thus required a large number of macrophages, the murine AM cell line MH-S was used. MH-S was chosen because this cell line has been demonstrated to be morphologically and functionally quite similar to its parent primary murine AMs [12]. A broad range of pathogen dose (0.001–1000 MOI) was tested to capture both the dose levels causing low to high immunological changes as well as the minimum dose(s) causing cellular damage.

Our results demonstrate that MI is a potent activator of inflammatory responses in the exposed alveolar macrophages (MH-S) in culture. MI activated the MH-S cells by inducing proinflammatory mediators including NO and inflammatory cytokines in a dose- and time- dependent manner, albeit to a variable extent. Interestingly, while the entire test dose range (0.001 – 1000 MOI) induced cellular and/or inflammatory/immunological responses, only the higher doses (10–1000 MOI) caused significant (p≤0.05) immunological response.

MI caused an increase in the proinflammatory cytokines TNF-α, IL-6, IL-1α, and IL-1β in the infected macrophages. The induction was observed as early as 3h post- infection, and the levels increased with time through the entire 24h post-infection period. The induction was dose-dependent, which may be due to a greater intracellular build up in macrophages of the responsible MI virulence factor or antigen at higher dose (MOI). Proinflammatory cytokine elevation in alveolar macrophages on exposure to MWF-isolated mycobacteria is particularly interesting, considering the available evidences of increased proinflammatory and decreased anti-inflammatory cytokines expression in hypersensitivity pneumonitis [10], [11], [15]. Inflammatory cytokines examined in this study, are known to be immune-regulatory molecules. For example, a high level of TNF-α is a crucial factor for controlling primary infection, as it induces the expression of other proinflammatory cytokines such as IL-1 and of several chemotactic cytokines, which attract immune cells to the site of infection [16], [17]. TNF-α in particular, as well as IL-1β and IL-6, are involved not only in innate immunity but also may regulate activation of other components of the immune response system, especially T cells and the macrophages (possibly in an autocrine manner).

Interestingly, IL-β and IL-1α were detected primarily in cell lysates, indicating their intracellular localization. Proinflammtory stimuli induce expression of the IL-1β proform but maturation and release are controlled by inflammasome that mediates caspase-1 dependent processing of IL-1β [18]. Caspase-1 and the inflammasome components are important in the host defense against pathogenic microorganisms as lack of caspase-1 in knock-out mice leads to an increased susceptibility to a variety of infections, such as those with *Francisella tularensis* [19], *Legionella pneumophila* [20], *Shigella* spp. [21], *Salmonella* spp. [22], [23] and *Pseudomonas aeruginosa* [24]. Experimental infections with some of these pathogens have been also investigated in knock-out mice lacking components of the inflammasome. In this respect, ASC-deficient mice have been shown to be more susceptible to infections with some bacteria (Francisella and Staphylococcus) [19], [25], as well as influenza viruses [26], demonstrating its importance in host defense mechanisms. In light of these observations in other pathogenic species of bacteria, MI 700506 might be involved in dysregulation of inflammosome activity in alveolar macrophages leading to intracellular localization of IL-1β.

Unlike the proinflammatory cytokines, IL-10 was below the detection limit in the infected MH-S cells. IL-10 is an anti-inflammatory and immunosuppressive cytokine and an inhibitor of activated macrophages and, as such, controls innate as well as cell-mediated immunity. IL-10 blocks the production of proinflammatory cytokines, such as IL-12 and TNF-α, and reduces the expression of MHC class II molecules, which are required for antigen presentation [27]. Our data suggest that MI inhibits IL10 expression in macrophages thereby allowing the activation of macrophages via induction of proinflammatory cytokines. Inability to control activated inflammatory response leads to lung cellular influx and neutrophil recruitment, which are a hallmark for HP pathology [28].

Substantial amount of NO was induced in the MI-infected macrophages which is a classical sign of inflammatory response. The NO production increased in a dose- and time- dependent manner. Proinflammatory cytokines particularly IL-6, TNF-α and IL-1β, are known to initiate the production of NO [29]. Although the cytotoxicity due to MI was prominent only with high doses and extended time of exposure, the initiation of the signaling cascade leading to cell death (apoptosis) maybe expected at the preceding lower doses in this model.

Mechanistic basis of the observed variable activation potential of individual genotypes toward macrophages is not yet clear. The genotypic differences may involve variable regulation of specific signaling and transcriptional machinery in response to variable repertoire of antigens or virulence factors in individual MI *g*enotypes.

Induction of proinflammatory cytokines in response to invading pathogens may result via activation of different host-cell signaling cascades and pathways. The p38 and JNK kinases are the members of the MAPK superfamily which plays important roles in signal transduction in a wide range of biological processes such as inflammation, cell survival, and apoptosis [30], [31]. We, therefore, examined the role of JNK and p38 in MI-infected macrophages. Our data showed that inhibition of either JNK or p38 could significantly block the cytokine induction (IL-1α, IL-1β, TNFα and IL-6) in MI-infected macrophages. This seems consistent with earlier studies on other cell-stimuli interactions [32], [33]. Previous studies have shown that species of mycobacteria trigger MAPK signaling pathways through engagement of TLRs [34], [35], [36], [37], [38] and that MAPK activation is required for mycobacteria-induced TNF-α secretion in certain species [34], [38] and cell death [39], [40], [41]. In this context, our results on dose-dependent upregulation and/or phosphorylation of both p38 and JNK in MI-infected macrophages imply the correlation between MAPK activation and inflammatory response. On the other hand, our MAPK inhibitor studies demonstrated that p38 and JNK pathways are necessary for the observed induction of different cytokines (IL-1α, IL-1β, TNF-α and IL-6) whereas GM-CSF appeared to be regulated via JNK pathway. These observations on role of p38 and JNK are in contrast with the observed role of ERK in cytokine induction by infected macrophages for other mycobacterial species [38], [41]. Our observation on significant alleviation of the viability loss in the infected cells treated with p38 and JNK inhibitors could possibly be ascribed to concomitant significant decrease in NO production in inhibitor-treated cells; this is because lowered NO can directly promote survival/multiplication of the pathogen in the MH-S cells leading to enhanced loss of viability. Alternately, there are several recent reports that also suggest that JNK and p38 MAPK pathways could play role in cell survival during stress [42], [43], [44] or infection [45] .

In conclusion, our data show that *M. immunogenum* induces inflammatory and cytotoxicity responses in alveolar macrophages in a dose- and time-dependent manner. Two of its genotypes, 700506 and MJY-3, cause greater proinflammatory changes as compared to the other known genotypes (MJY-4, MJY-12 and MJY-14). Variable induction of proinflammatory cytokines may account in part for the differential HP-inducing potential of these MI genotypes. To gain a better understanding of the antigenic proteins or virulence factors involved in the activation of alveolar macrophages, genetic disruption of bacterial factors might be useful. The results further show that the inflammatory responses are primarily mediated via p38 and JNK suggesting the role of MAPK pathway in MI-caused HP immunopathogenesis. Follow-up studies in this direction will elucidate further mechanisms by which *M. immunogenum* is able to regulate/activate MAPK and other host-signaling pathways for establishing HP pathology. The current study on the interactions between *M. immunogenum* and host lung macrophages is a significant initial step in the direction of our emerging understanding of the immunopathogenesis mechanisms of occupational HP in machinists.
